# Same structure, different mechanisms?

**DOI:** 10.7554/eLife.56501

**Published:** 2020-04-23

**Authors:** Francis TF Tsai, Christopher P Hill

**Affiliations:** 1 Department of Biochemistry and Molecular Biology, Baylor College of MedicineHoustonUnited States; 2 Department of Biochemistry, University of Utah School of MedicineSalt Lake CityUnited States

**Keywords:** AAA+ protease, protein translocation, protein unfolding, *E. coli*, *Neisseria meningitidis*

## Abstract

Two interpretations of similar structures for the same molecular machine illustrate the limits of inferring biochemical mechanism from protein structure.

**Related research article** Ripstein ZA, Vahidi S, Houry WA, Rubinstein JL, Kay LE. 2020. A processive rotary mechanism couples substrate unfolding and proteolysis in the ClpXP degradation machinery. *eLife* **9**:e52158. doi: 10.7554/eLife.52158**Related research article** Fei X, Bell TA, Jenni S, Stinson BM, Baker TA, Harrison SC, Sauer RT. 2020. Structures of the ATP-fueled ClpXP proteolytic machine bound to protein substrate. *eLife* **9**:e52774. doi: 10.7554/eLife.52774

Pursuit of the Francis Crick dictum, ‘if you want to understand function, study structure’ has driven remarkable advances in understanding of protein mechanisms (Crick, 1988). However, there are always limits.

A classic goal in structural biology has been to understand AAA+ ATPases, a large and diverse family of enzymes that perform mechanical work for cells (Harrison, 2004). A group of AAA+ ATPases called unfoldases use the energy of ATP hydrolysis to unfold protein substrates, apparently by pulling the substrate through the central pore of the ring-shaped enzyme. The debate around how these unfoldases work has been enlivened by two recent reports in eLife, one by John Rubinstein, Lewis Kay and colleagues at the University of Toronto – including Zev Ripstein and Siavash Vahidi as joint first authors (Ripstein et al., 2020) – and another by Robert Sauer and colleagues at the Massachusetts Institute of Technology and Harvard Medical School – including Xue Fei as first author (Fei et al., 2020). The two groups report very similar structures of the ClpX unfoldase in complex with ClpP, an enzyme that breaks down protein substrates unfolded by ClpX (Figure 1). The complex formed is referred to as the ClpXP protease. Like most AAA+ ATPases, ClpX is a hexamer, whereas ClpP displays seven-fold symmetry. Despite the close similarity of the ClpXP structures reported, the two groups propose very different mechanisms of action for the unfoldase.

**Figure 1.**
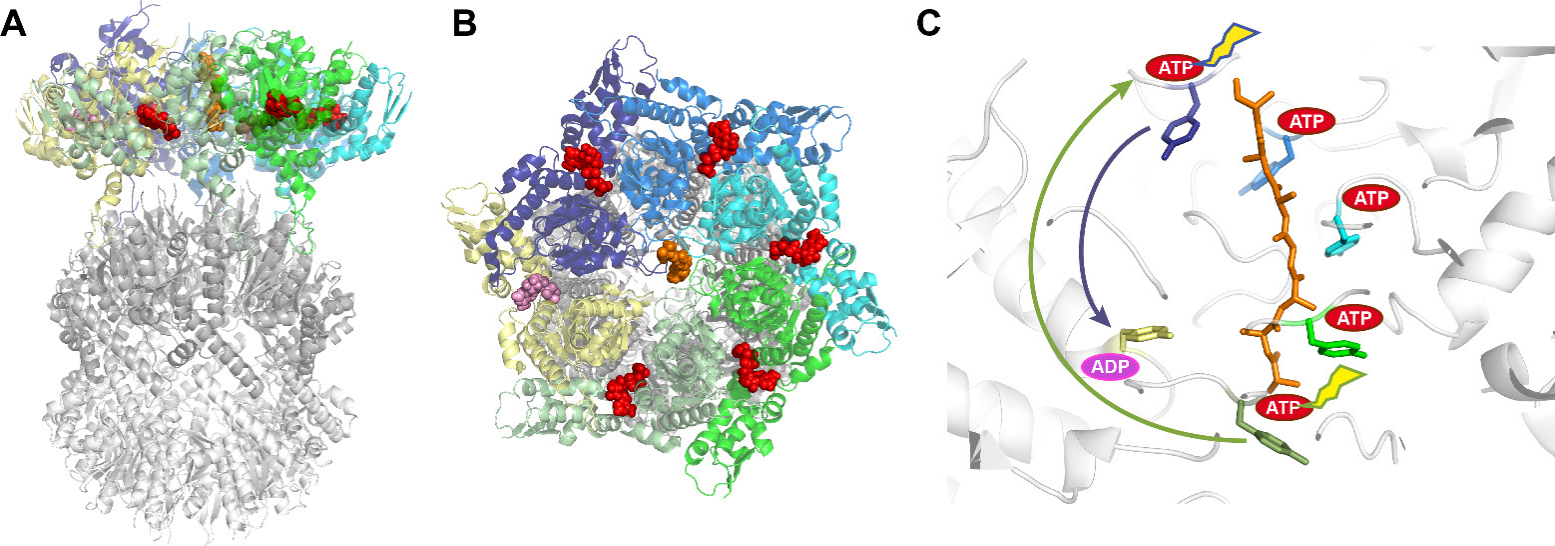
Structure of the ClpXP complex. (**A**) Side and (**B**) top-down views of the ClpXP complex bound to a substrate (shown in orange). The six ClpX subunits are each shown in a different color (purple, blue, cyan, green, sage green, and yellow), with ATP shown in red and ADP in pink. The same colors are used throughout the figure. (**C**) Close-up side view of the pore region in ClpX with arrows indicating the proposed sequential (sage green arrow; Ripstein et al., 2020) and probabilistic (purple arrow; Fei et al., 2020) mechanisms. The tyrosines lining the ClpX pore are colored according to their corresponding ClpX subunit. Five of these tyrosines bind the substrate while the tyrosine of the yellow subunit, which is bound to ADP, does not contact the substrate. The ATP hydrolyzed in each of the models is indicated by a flash. In the sequential model (Ripstein et al., 2020), ATP hydrolysis in the lower subunit allows it to disengage from substrate, transition through the ‘yellow state’, exchange ADP for ATP, and bind the next two residues of the substrate by docking against the top-most subunit. Because each subunit binds two residues, the net result is ClpX ‘walking’ up the substrate and translocation of two substrate residues down toward ClpP. In the probabilistic model (Fei et al., 2020), ATP hydrolysis at the top position (sometimes preceded by hydrolysis at other positions) causes the upper subunit to maintain a tight grip on substrate and move it down toward the ‘yellow state’, thereby translocating substrate by approximately six residues toward ClpP.

A flurry of AAA+ unfoldase structures bound with substrate or substrate mimics has been reported over the past three years (Gates and Martin, 2020). These structures show unfoldase subunits arranged in a spiral stabilized by ATP. In some cases, all six subunits participate in the spiral, but in most cases one or more subunits seem to be disengaged, as if moving from one end of the spiral to the other.

A sequential ‘hand-over-hand’ mechanism has been proposed for a majority of these unfoldase structures. In this model, one or more subunits of the unfoldase bind the substrate along with a molecule of ATP as they move sequentially from one end of the spiral to the other. ATP hydrolysis at the bottom of the spiral allows the bottom-most subunit to move to a transitioning position and release the substrate. Subsequent exchange of ADP for ATP allows this transitioning subunit to rejoin the top end of the spiral and bind the next two residues of the substrate (Figure 1C). This way, ATP binding and hydrolysis proceed sequentially around the AAA+ hexamer and, depending on the frame of reference, the consequence can be described as the unfoldase walking along the substrate or as the substrate being pulled through the unfoldase pore. From either perspective, two amino acid residues of the substrate are translocated for each ATP hydrolyzed.

Ripstein et al. and Fei et al. report structures of ClpXP complexes from *Neisseria meningitidis* and *Escherichia coli,* respectively. The two structures are very similar to each other and the ClpX hexamer closely resembles multiple other AAA+ unfoldases (Gates and Martin, 2020). The structures also show how the hexameric ClpX binds the heptameric barrel-shaped ClpP protease to align the substrate with the opening to the chamber in ClpP where the substrate is broken down. However, the two groups have proposed different mechanisms for the unfoldase. Ripstein et al. favor the two-residue step hand-over-hand mechanism. In contrast, Fei et al. propose a radically different model in which ATP hydrolysis at the top of the spiral allows the top-most ClpX subunit to retain its grip on the substrate. The subunit then moves to the bottom of the spiral, pulling the substrate approximately six amino acid residues towards ClpP before releasing it (Figure 1C).

The model proposed by Ripstein et al. has several attractive features. First, it is similar to analogous mechanisms of more distantly related nucleic acid translocases (Lyubimov et al., 2011; Enemark and Joshua-Tor, 2008). Second, it explains how widely divergent substrate sequences can be bound and processed. And third, the asymmetric nature of the spiral indicates multiple structural steps along the proposed reaction cycle. Balanced against this, the model of Fei et al. also has attractive features. First, it is consistent with published estimates of the step size (Olivares et al., 2016). And second, by also allowing ATP hydrolysis to occur at subunits within the spiral, it explains how multiple inactive ATPase sites can be accommodated (Martin et al., 2005).

Together, these two studies, along with another recent study that reported a different structure for ClpXP (Gatsogiannis et al., 2019), add new fuel to a debate that seemed settled. Key questions for future studies include: Do different AAA+ unfoldases use the same mechanism to couple ATP hydrolysis with substrate translocation? Are the mechanisms sequential? Where and how is ATP hydrolysis triggered? How might one ADP-bound subunit in the model of Fei et al. bind a substrate tightly enough to enable translocation? And, how many amino acid residues are translocated per ATP hydrolyzed? Moreover, the additional proposal from Ripstein et al. that ClpX and ClpP rotate with respect to each other during the reaction cycle seems likely to provoke further controversy. Just when AAA+ unfoldases seemed to have yielded their mechanistic secrets, these two new structures look very much as expected but nevertheless create a host of new questions.
